# Direct and indirect costs of paediatric asthma in the UK: a cost analysis

**DOI:** 10.1136/archdischild-2023-326306

**Published:** 2024-05-27

**Authors:** Charlotte T Kennedy, Graham S Scotland, Seonaidh Cotton, Stephen W Turner

**Affiliations:** 1 Health Economics Research Unit, University of Aberdeen, Aberdeen, UK; 2 Centre for Healthcare Randomised Trials, University of Aberdeen, Aberdeen, UK; 3 Child Health, Royal Aberdeen Children's Hospital, Aberdeen, UK

**Keywords:** Health Care Economics and Organizations, Child Health, Respiratory Medicine, Paediatrics

## Abstract

**Objective:**

To estimate the cost of paediatric asthma from a UK National Health Service (NHS) and societal perspective and explore determinants of these costs.

**Design:**

Cost analysis based on data from a large clinical trial between 2017 and 2019. Case report forms recorded healthcare resource use and productivity losses attributable to asthma over a 12-month period. These were combined with national unit cost data to generate estimates of health service and indirect costs.

**Setting:**

Asthma clinics in primary and secondary care in England and Scotland.

**Main outcome measures:**

Cost per asthma attack stratified by highest level of care received. Total annual health service and indirect costs. Modelled effect of sex, age, severity, number of attacks and adherence on total annual costs.

**Results:**

Of 506 children included in the analysis, 252 experienced at least one attack. The mean (SD) cost per attack was £297 (806) (median £46, IQR 40–138) and the mean total annual cost to the NHS was £1086 (2504) (median £462, IQR 296–731). On average, children missed 6 days of school and their carers missed 13 hours of paid work, contributing to a mean annual indirect cost of £412 (879) (median £30, IQR 0–477). Health service costs increased significantly with number of attacks and participant age (>11 years). Indirect costs increased with asthma severity and number of attacks but were found to be lower in older children.

**Conclusions:**

Paediatric asthma imparts a significant economic burden on the health service, families and society. Efforts to improve asthma control may generate significant cost savings.

**Trial registration number:**

ISRCTN 67875351.

**WHAT IS ALREADY KNOWN ON THIS TOPIC**

- Direct and indirect costs of paediatric asthma in the UK have not been estimated for many years.
- Older data indicate that costs to the health service are higher for those with more severe and uncontrolled asthma due to an increased requirement for hospital care.
- The condition is associated with considerable indirect costs arising from days missed from school and carers having to take time off work to care for children.

**WHAT THIS STUDY ADDS**

- This study provides detailed contemporary healthcare resource use and productivity loss estimates resulting from paediatric asthma over a 1-year period, and quantifies their relationship with patient and disease characteristics.
- Annual costs to the health service are more than threefold higher for children who experience three to four attacks compared with those with no attacks.
- Productivity losses are 1.7 times higher for families of participants with more severe (British Thoracic Society (BTS)/Scottish Intercollegiate Guidelines Network (SIGN) step 4) versus less severe (BTS/SIGN step 2) disease, and 6.7 times higher for those who experience three to four attacks per year compared with no attacks.

**HOW THIS STUDY MIGHT AFFECT RESEARCH, PRACTICE OR POLICY**

- This study highlights the potential economic savings of improved asthma treatment to reduce the number of attacks.
- The estimates of resource use and cost can serve as key inputs to service planning and future economic modelling studies of asthma interventions and initiatives.

## Introduction

Asthma affects between 5% and 10% of children in the UK.^1–3^ Asthma attacks are common, can be life threatening and impose a considerable economic burden on the health service, families and society.^4–7^ It has been estimated that provision of care for all patients with asthma costs the National Health Service (NHS) £3.03 billion per year.^6^

The cost of asthma is known to be associated with severity and control; more severe and less controlled asthma is linked to the greatest healthcare resource use (HCRU).^8–10^ There is a lack of recently published studies that have quantified the economic burden that paediatric asthma imposes on the health system and society in the UK. This may limit the ability of planners and commissioners to accurately budget for the delivery of asthma services or determine the potential value of initiatives to improve its control. To address this evidence gap, we use data collected from a randomised controlled trial (RCT) which recruited children with asthma between 2017 and 2019 to describe the direct and indirect costs of childhood asthma in the UK.

## Methods

### Study design

The Reduced Asthma Attacks in Children using Exhaled Nitric Oxide (RAACENO) trial recruited 509 children from centres across the UK between 2017 and 2019, the majority (97%) from secondary care. Participants were aged 6–15 years, diagnosed with asthma, treated with inhaled corticosteroids and had at least one asthma attack in the past year. The trial found no evidence for a difference in clinical outcomes or costs between the randomised treatment groups (FeNO plus symptom-guided treatment or symptom-guided treatment alone), justifying the pooling of data for this cost analysis. Full details of the trial results, objectives and design are available elsewhere.^11^

### Data collection

Data were collected retrospectively from parent-held diaries and case report forms (CRFs) completed during four clinic assessments at 3-month intervals throughout the follow-up period. The information relevant to this analysis included asthma medications, treatment adherence, number of asthma attacks and associated healthcare use, other asthma-related healthcare use and participant and carer time lost from usual activities.

### Cost estimation methods

The analysis took a UK NHS perspective for HCRU and a societal perspective for time losses. All costs are expressed in 2019/2020 UK sterling using the NHS inflation indices where necessary.^12^ The costs incurred during each quarterly time interval were summed at the individual level across all observations over the 1-year follow-up period.

Three categories of asthma-related HCRU were identified from the CRFs: (1) HCRU associated with attacks; (2) HCRU not associated with attacks; and (3) medication. Direct NHS costs under each category were calculated by multiplying the reported HCRU by nationally representative unit costs (online supplemental table 1).^12–14^ Staff time required for pharmacist and NHS 111 contacts were informed by external literature.^15 16^ The cost of medications was sourced from the British National Formulary (BNF) and combined with the prescribed dose (online supplemental table 2).^17^ An NHS confidential discount is available for omalizumab injections. For this analysis we used the list price.^18^

10.1136/archdischild-2023-326306.supp1 Supplementary data

Indirect costs were measured using the human capital approach.^19^ Time displaced was categorised as: school, leisure, study and paid or unpaid work. Details of the unit costs applied to time losses are presented in online supplemental material 1.

### Statistical analysis

Data were summarised using the mean, SD, median and IQR for continuous variables, and numbers and percentages for categorical variables. While healthcare cost data are often right skewed, whereby a minority of high-resource patients lift the mean above the median, the analysis and interpretation focuses on the mean as the most relevant measure of budgetary impact.^20^ All analyses were conducted using STATA V.15.^21^

Missing data were minimised by asking about patient-reported resource use and time losses at each attended clinical assessment visit, covering the time elapsed since the last attended visit. If patients failed to attend their final follow-up assessment, they were followed by phone to collect data on all attacks and associated resource use. We assessed the sensitivity of results to missing data using multiple imputation with chained equations.^22^

Generalised linear models (GLM) were used to explore the effect on total cost of several covariates: age, sex, asthma severity, number of attacks and treatment adherence. Age was categorised as per the minimisation criteria used in the RAACENO trial (<11 years; ≥11 years). Asthma severity was categorised by level of asthma medication using the British Thoracic Society (BTS) / Scottish Intercollegiate Guidelines Network (SIGN) step system.^23^ Adherence was measured on a scale between 0 and 1 (online supplemental material 2 and online supplemental table 3), with a value of ≥70% defined as adherent. The number of attacks was categorised as 0, 1–2, 3–4 or ≥5, to allow for non-linearity in the relationship between number of attacks and cost. The cost of study assessment visits was excluded from the GLM analysis of direct health service costs, as these were potentially protocol driven and invariable between patients. GLM results are expressed in terms of the average effect of each variable on the model’s predicted cost. Given the skewed nature of the data, gamma family and log link functions were specified based on findings of modified Park and Box-Cox tests of functional form.^24 25^ A high proportion of zero values were observed for indirect costs, which creates challenges for statistical modelling. A two-part model was specified with a probit model first used to predict the probability of reporting time losses combined with a GLM model to predict costs among those with time losses.^26^ These models are combined to estimate the effects of covariates on total indirect costs. Both models used robust SEs clustered by centre number. The trial was registered with the ISRCTN registry (ISRCTN 67875351).

## Results

### Baseline characteristics

There were 509 participants recruited of whom 506 were included in the analysis. We have previously shown that the RAACENO participants were comparable to children attending secondary care asthma clinics in the UK.^11^ Most patients (85%) were on step 3 or step 4 BTS/SIGN treatment levels and 56% had an inpatient admission for asthma in the year preceding recruitment (table 1).

**Table 1:** Baseline characteristics

		N	n/Mean	%/SD	Median	IQR
Sex, male (n, %)		506	306	60.5		
Age, years (mean, SD)		506	10.1	2.6	10	(8, 12)
	<11 (n, %)	506	285	56.3		
	≥11 (n, %)	506	221	43.7		
Age at diagnosis, years (mean, SD)		503	3.8	2.6	3	(2, 5)
Weight, kg (mean, SD)		502	39.9	16.4	36.7	(27.6, 48.6)
Centre, secondary (n, %)		506	490	96.8		
Treatment level* (n, %)						
	BTS/SIGN step 2	506	76	15.0		
	BTS/SIGN step 3	506	217	42.9		
	BTS/SIGN step 4	506	213	42.0		
Controlled† (n, %)		506	256	50.6		
Hospital admissions in the last year (n, %)						
	None	506	221	43.7		
	1–2	506	201	39.7		
	3–4	506	55	10.9		
	≥5	506	29	5.7		
Days absent from school in the last year (mean, SD)		504	15.0	14.4	10	(4.8, 20.0)
Oral steroid courses in the last year, count (mean, SD)		506	3.6	3.0	3	(1, 5)
Prescription details						
	LTRA (n, %)	506	303	59.9		
	Reliever inhaler (n, %)	506	506	100		

*Treatment levels based on the British Guideline on the Management of Asthma.^23^

†Asthma Control Test score ≥20.

BTS, British Thoracic Society; LTRA, leukotriene receptor antagonist; SIGN, Scottish Intercollegiate Guidelines Network.

### Direct cost to the health service

#### Attacks

There were 509 attacks observed in 252 participants during follow-up. Associated HCRU data were available for 244 participants (497 attacks), with 49 (61 attacks) requiring inpatient hospital admission. The mean cost per attack was £297 (SD: 806) and the median was £46 (IQR: 40, 138). Table 2 presents the cost per attack overall and by the highest level of care received. The average number of healthcare contacts is provided in online supplemental table 4. Most attacks (n=291) were treated in primary care, predominantly by a general practitioner. Where secondary care was required, participants were typically treated in the emergency department, followed by hospital inpatient admissions. No healthcare contact was made for 46 attacks (29 participants) which were treated with a course of oral steroids available at home. Results by treatment group are provided in online supplemental table 5.

**Table 2:** Healthcare cost associated with each attack stratified by highest level of care received

			All		Primary		Secondary		At home*	
Number of participants (N=244/252)†			244		171		115		29	
Number of attacks (N=497/509)‡			497		291		160		46	
			**Mean**	**SD**	**Mean**	**SD**	**Mean**	**SD**	**Mean**	**SD**
Total cost per attack			£297	806	£57	52	£819	1271	£1.41	2.63
	Primary care		£42	51	£55	52	£30	47		
		GP	£31	33	£42	27	£21	39		
		Nurse	£2.03	8.19	£2.09	8.42	£2.52	8.87		
		NHS 24/111	£1.07	4.92	£1.07	5.97	£1.38	4.01		
		Out-of-hours GP service	£5.66	37	£7.63	47	£3.70	18		
		Walk-in centre	£1.75	9.69	£2.36	11	£1.14	7.16		
		Pharmacist	£0.03	0.43	£0.02	0.40	£0.04	0.54		
	Secondary care		£253	812			£787	1277		
		Emergency department	£23	54			£71	74		
		Hospital outpatient	£4.21	26			£13	45		
		Hospital inpatient	£220	814			£684	1323		
		Day case	£1.58	25			£4.92	44		
		Ambulance	£4.56	37			£14	64		
	Medication		£1.65	1.95	£1.66	1.81	£1.71	1.99	£1.41	2.63

*One-week course of 5 mg prednisolone per day.

†N=patients with attacks with complete resource use data/observed number of patients with attacks.

‡N=attacks with complete resource use data/observed attacks.

GP, general practitioner; NHS, National Health Service.

#### Total HCRU

Besides attack-related HCRU and study follow-up assessments, a large proportion of participants (n=225/443) did not report any additional HCRU. Table 3 reports the mean total cost of asthma-related HCRU over the trial follow-up period. The mean cost of asthma is driven by participants who had multiple attacks, required inpatient hospitalisation and were prescribed high-cost omalizumab (n=13/432). All participants used a preventer (inhaled corticosteroid±long-acting beta agonist) and reliever inhaler. Other medication use included leukotriene receptor antagonist (n=287/432), theophylline (n=50/432) and ciclosporin (n=6/432). A detailed breakdown of cost by treatment group and corresponding HCRU is found in online supplemental tables 6 and 7.

**Table 3:** Total healthcare resource use costs observed during trial follow-up

Category (patients contributing data (N))		Patients reporting any resource use (n (%))	Mean*	SD	Median*	IQR
Total including clinical study visits (N=430)†		430 (100)	£1846	2514	£1230	(1055, 1518)
Total excluding clinical study visits (N=430)		430 (100)	£1086	2504	£462	(296, 731)
Attack related (N=498)		244 (49.0)	£604	1284	£140	(57, 393)
	Primary care	206 (41.4)	£101	99	£79	(40,119)
	Secondary care	115 (23.1)	£1095	1698	266	(133, 1906)
	Oral steroid	244 (49.0)	£3.36	4.45	£2.11	(0.36, 4.78)
Not associated with attacks (N=443)		218 (49.2)	£295	650	£133	(40, 250)
	Primary care	178 (40.2)	£90	90	£50	(40, 114)
	Secondary care	102 (23.0)	£476	888	£204	(133, 408)
	Medication‡	4 (0.9)	£1.75	0.00	£1.75	(1.75, 1.75)
Medication for asthma management (N=432)		432 (100)	£647	2092	£345	(234, 433)
	Immunoglobulin injections (omalizumab)	13 (3.0)	£10 824	5592	£11 150	(4520, 14 736)
Clinical study visits (N=506)†		481 (95.0)	£721	167	£816	(612, 816)

*Cost for those incurring any resource use (excluding those with zero cost)*.*

†Quarterly clinical study visits were excluded from the regression analysis of cost determinants.

‡In addition to medication prescribed at regular monitoring visits for the participant’s asthma.

### Indirect cost

Table 4 summarises participant-reported time losses from productive activities and corresponding indirect costs for those reporting time losses. Most participants reported missing days of school due to asthma. Approximately half (52%) of adult family and carers reported time losses, mostly from paid employment. The mean indirect cost in the population, inclusive of those reporting zero time losses, was £412 (SD: 879). The median was £30 (IQR: 0, 477). On average, participants reported missing 5.9 (SD: 8.5) days of school. Results by treatment allocation group are provided in online supplemental table 8.

**Table 4:** Indirect costs of asthma captured over the trial follow-up

Category (patients contributing (N))		Patients reporting any time losses (n (%))	Mean*	SD	Median*	IQR
Days displaced (child) (N=443)		333 (75.2)	8.2	9.2	5	(2, 11)
	School	332 (74.9)	7.9	9.0	5	(2, 11)
	Paid employment	45 (10.2)	2.6	2.3	2	(1, 3)
Hours displaced (adults†) (N=443)		225 (50.8)	56	85	26	(12, 60)
	Paid employment	161 (36.3)	36	42	20	(8, 44)
	Unpaid work	103 (23.3)	45	71	18	(7, 56)
	Leisure	58 (13.1)	29	51	8	(4, 25)
	Study	24 (5.4)	21	29	8.5	(4, 23)
Indirect costs (child)‡ (N=443)		45 (10.2)	£90	80	£70	(35, 104)
Indirect costs (adults†) (N=443)		219 (49.4)	£816	1096	£470	(159, 936)
	Paid employment	161 (36.3)	£709	837	£397	(159, 874)
	Unpaid work	103 (23.3)	£543	854	£216	(84, 673)
	Leisure	58 (13.1)	£148	257	£40	(20, 126)
Total cost (N=443)		229 (51.7)	£798	1090	£451	(159, 914)

*Cost reported for those incurring any resource use (excluding those with zero cost).

†Any adult relative or carer of the participant.

‡Does not include indirect costs associated with time losses from education.

### Determinants of total direct and indirect costs

The results from GLMs of direct and indirect costs are presented in table 5, in terms of the effect of each explanatory variable on modelled cost. The direct cost model predicts costs of £2841 (SE: 658) for patients ≥11 years old with more severe asthma (BTS step 4) who experience ≥5 attacks in the year. Patients <11 years old, with less severe asthma (BTS step 2), who experience no attacks, are predicted to cost the NHS £521 (SE: 137). A similar pattern was observed for indirect cost, but with a negative association for age. The probability of reporting no time losses was 0.65 (SE: 0.04, p<0.01) if the participant experienced no attacks.

**Table 5:** Results of the GLM model of direct health service costs and the two-part model of indirect costs presented in terms of predictive margins and average marginal effects

Covariates		Direct cost (N=430)					Indirect cost (N=443)				
		Predictive margins	SE	Marginal effect	SE	P value*	Predictive margins	SE	Marginal effect	SE	P value*
Predicted cost		£1104	250				£415	45			
Sex											
	Male	£1208	340				£405	60			
	Female	£961	146	−£247	235	0.20	£431	53	£26	73	0.86
Age (years)											
	<11	£885	162				£509	64			
	≥11	£1423	425	£538	303	<0.01	£293	50	−£217	74	<0.01
Treatment level†											
	BTS/SIGN step 2	£974	236				£275	52			
	BTS/SIGN step 3	£1122	431	£148	426	0.72	£393	51	£118	66	0.06
	BTS/SIGN step 4	£1113	153	£139	272	0.62	£465	80	£191	87	<0.01
Number of attacks											
	0	£736	259				£144	28			
	1–2	£1138	262	£402	122	<0.05	£504	60	£361	64	<0.01
	3–4	£2302	588	£1565	562	<0.01	£985	185	£841	185	<0.01
	≥5	£2188	434	£1452	417	<0.01	£1114	289	£970	281	<0.01
Adherence											
	Not adherent (<70%)	£807	86				£317	60			
	Adherent (≥70%)	£1271	354	£464	297	<0.05	£464	54	£146	70	0.11

*P value from generalised linear model (GLM) regression*.*

†Treatment levels based on the British Guideline on the Management of Asthma.^23^

BTS, British Thoracic Society; SIGN, Scottish Intercollegiate Guidelines Network.

The mean adherence was 72.5% (SD: 20.0). Adherence was found to be predictive of direct cost (p=0.03); adherent patients were projected to cost £464 (SE: 298) more than non-adherent. Previous asthma studies^27 28^ defined ‘adherent’ as ≥80%. Under this definition, adherence was not found to be a predictor of direct cost (p=0.93). The estimates were robust to missing data assumptions, with similar results obtained using multiple imputation. In a further sensitivity analysis which removed participants who received omalizumab, the effects of age and adherence on direct costs were no longer significant, and the predicted annual direct costs were substantially lower (£728) (online supplemental table 9).

## Discussion

This study provides detailed estimates of the cost of paediatric asthma from both the NHS and societal perspective, for a population prone to attacks, followed up mainly in secondary care. The cost per attack to the health service (£297) was driven by hospital inpatient admissions, despite only 12% of attacks leading to admission. The mean cost to the health service was £1086 per participant over the 12-month follow-up period, inclusive of £647 for medication, £296 of attack-related resource use and £145 for other resource use. Participants missed on average 5.9 (SD: 8.5) days of school and parents and carers missed 13.0 (SD: 30.6) hours of work in relation to the child’s asthma. These findings highlight the often unrecognised costs of asthma to children and families.

The higher direct cost for older relative to younger children and the unexpected finding of higher cost for adherent compared with non-adherent children can be explained by omalizumab prescribing being clustered in more adherent children over the age of 11, at a cost of up to £18 078 per year based on the list price. Just 13 participants received this medication, 10 of whom were >11 years old and had adherence >70%. Adherence and age were no longer predictive of direct costs when these participants were removed from the analysis. The finding of lower indirect costs for older participants may be explained by families becoming more knowledgeable of the condition as children age, and therefore better able to manage it without taking time off work. We also know that severity and control improve as children age,^29 30^ so we may be observing a mediation between asthma severity and age.

A strength of this analysis is that it is based on data from a large multicentre RCT conducted in England and Scotland. A high degree of complete data was achieved, particularly in relation to attack-related resource use. The number of attacks, hospitalisations and school days missed were substantially higher in the year preceding randomisation. It is unclear if such improvement would be achieved outside the trial setting, meaning that ‘real world’ costs might be higher. Data may be subject to protocol biases as participants attended clinical assessments more regularly than in routine practice and may have had better control as a result. It would be of value to externally validate these findings using observational data. Nevertheless, our estimated cost per attack, and effects of covariates on direct and indirect costs, should be generalisable to the wider paediatric asthma population. A further weakness is that our study did not collect information regarding attack severity. There is scope to better understand the determinants of attack cost to the NHS given the high variability observed. There are also limitations of how adherence was measured and defined, with some discrepancies between different sources leading to uncertainty regarding its accuracy. Finally, our estimate of indirect costs is restricted to productivity losses arising from absenteeism. It does not capture presenteeism so may underestimate the full indirect costs of asthma.

There is a paucity of contemporary studies reporting on the cost of paediatric asthma in the UK.^8 10^ Kerkhof *et al* reported a mean annual HCRU cost of £861 (2015 prices) for patients with severe, uncontrolled eosinophilic asthma. Our estimate for secondary care managed paediatric asthma, inclusive of quarterly clinical assessment visits, is higher at £1846. This may be explained by differences between the study populations—only 2% of the population reported by Kerkhof *et al* were under 18 years of age—and more intensive routine monitoring within the RAACENO trial. Gokhale *et al* 8 assessed the HCRU of 265 paediatric asthma patients aged 6–17 years in England who had severe refractory asthma. During a 1-year period, 42.6% of patients experienced at least one oral corticosteroids (OCS)-defined attack (excluding attacks treated at home), and 24.5% of attacks resulted in a hospital attendance. In RAACENO, we found that 48.6% (242/498) of participants experienced at least one attack requiring contact with primary or secondary care, with 35.5% of these resulting in secondary care contact.

Our results with respect to productivity losses are broadly consistent with those reported in the literature. Based on a review of published evidence, Nunes *et al* reported that a child experiencing an exacerbation can expect to miss 3–5 days of school, with parents missing a similar number of days of work.^5^ RAACENO participants experienced two attacks on average. Those who experienced at least one attack reported missing 9.4 (SD: 10.3) days of school and their carers reported missing 20.9 (SD: 39.2) hours of paid work

The results of this study can support the planning of asthma services for similar populations. This is directly relevant to initiatives to improve asthma care and outcomes in the NHS such as phase 1 of the National Bundle of Care for Children and Young People with Asthma.^31^ It is estimated there are currently 1.1 million children receiving treatment for asthma in the UK.^32^ A recent UK-based cohort study of children with asthma in primary care suggests an annual attack rate of at least 0.18 per patient-year.^33^ Assuming our cost per attack estimate is generalisable to attacks experienced by the wider paediatric asthma population, the expected annual cost of asthma attacks would be in the region of £59.2 million at the population level ( 1.1M×0.18×£297) ). Acknowledging the caveats around generalisability of hospitalisation rates for attacks observed in the RAACENO study, this simple calculation illustrates how our results could be used to inform service planning and future modelling initiatives.

In conclusion, paediatric asthma imparts a significant economic burden on the health service, families and society. Our data provide a useful input to future studies that seek to model the broader costs and savings of efforts to improve paediatric asthma control and reduce attack frequency.
