# Supplementary material for: Direct and indirect costs of paediatric asthma in the UK: a cost analysis
Source: Arch Dis Child. 2024 May 27;109(9):724–9. doi: 10.1136/archdischild-2023-326306 (PMC11347193; doi:10.1136/archdischild-2023-326306)
Supplement: Supplementary data [file archdischild-2023-326306supp001.pdf]

The direct and indirect cost of paediatric asthma in the UK: a cost analysis

Supplemental material

Contents

Supplemental material 1: Calculation of indirect costs and outcomes 1

Supplemental material 2: Calculation of adherence score 1

Supplemental tables 2

Supplemental table 1: Unit cost of health care contacts 2

Supplemental table 2: Unit cost of medication sourced from the British National Formulary for Children 4

Supplemental table 3: Decision rules for participant's adherence score 6

Supplemental table 4: Healthcare resource use contacts associated with each attack stratified by highest level of care received.[1] 6

Supplemental table 5: Healthcare resource use cost associated with each attack by treatment arm (N=497/509[1]) 8

Supplemental table 6: Total healthcare resource use contacts observed during trial follow-up[1] 9

Supplemental table 7: Total healthcare resource use cost observed during trial follow up by treatment arm[1] (N=443/506) 11

Supplemental table 8: Indirect cost of asthma captured over the trial follow-up by treatment arm (N=443/506) 13

Supplemental table 9: Results of the GLM model of direct health service costs including and excluding patients prescribed omalizumab (n=13) during trial follow-up 14

References 15

The direct and indirect cost of paediatric asthma in the UK: a cost analysis

Supplemental material 1: Calculation of indirect costs and outcomes

A cost per hour was applied dependent upon the activity reported. The value of time forgone by carers from for paid employment was determined using a weighted average of the age distribution of the parents of primary school age children (1) against the gross average wage rates by age from the Annual Survey of Hours and Earnings (ASHE). (2)Time lost from unpaid work for carers was valued using the average cost per hour of housework, volunteering and informal care published by the Office for National Statistics (ONS).(3) The value of time lost from leisure activities was valued using the current value of non-working time published by the Department of Transport.(4) Time lost from paid employment for participants was estimated using an 8-hour workday multiplied by the national minimum wage.(5)

We did not apply a unit cost for time losses from education (i.e., study or school days), as there is no accepted monetary value. Therefore, we present educational losses in unvalued units of time.

Supplemental material 2: Calculation of adherence score

Asthma management medication (i.e., ICS inhalers) adherence was measured using a variety of factors during the trial. This was through smart inhaler devices, research nurse opinion and family reported adherence at quarterly trial follow up visits. All adherence outcomes were reported in the trial Case Report Forms (CRFs).

The smart inhaler records daily adherence as a percentage measure – where a score ≥70% was defined as “adherent” in the trial. However, several issues were found with the smart inhaler measurement. (6) Firstly, it was not possible to capture the adherence data where follow-up visits were carried out over the phone. Secondly, missing data issues were also observed where the participant would forget the inhaler at their follow-up visit. Finally, the smart inhaler score was not always consistent with the other two measures of adherence: research nurse opinion and family reported adherence. The discordance between measures can be attributed to technical issues of the device in recording and uploading the data and user issues where participants had two smart inhalers or where the device was insufficiently charged for example.

Therefore, to calculate a score of adherence we constructed an algorithm which takes all three measures to alleviate issues where the smart inhaler under-estimated true adherence (Online supplementary material table 3). Using this measure, it is possible that where a participant scores 0% on the smart inhaler, they can still score 100% if the family and clinical team are of the opinion the participant used their inhaler “all of the time”.

The direct and indirect cost of paediatric asthma in the UK: a cost analysis

Supplemental tables

Supplemental table 1: Unit cost of health care contacts

Resource use		Type of contact	Unit cost	Source
**Primary care**				
GP		Visit to surgery	£39.65	PSSRU 2019 (£4.30 per minute of staff time at average consultation length of 9.22 minutes)(7,8)
		Phone	£23.22	PSSRU 2019 (£4.30 per minute of staff time at average phone consultation length of 5.4 minutes) (7,8)
Community asthma nurse		Visit to surgery	£28.00	PSSRU 2019[69] (£84 per hour of participant contact of band 6 GP nurse at average Nurse consultation visit length of 20minutes)(7)
		Home visit	£23.14	PSSRU 2010 (Inflated to 2019 prices)(9)
		Phone	£7.97	PSSRU 2019 (£84 per hour of participant contact of band 6 GP nurse at average nurse phone consultation length of 5.69 minutes) (7,8)
	NHS 24/111		£12.96	Pope, Turnbull et al. (2017). £12.26 inflated to 2019 prices using the PSSRU inflation indices.(7,10)
	Out of hours GP service		£74.02	Weighted average of T03A & T03NA (excluding emergency dental), NHS reference costs 2018/19.(11)
	Walk-in centre		£45.71	Weighted average of T04A & T04NA (excluding emergency dental), NHS reference costs 2018/19. (11)
	Pharmacist		£6.82	9.22 minutes of band 6 community-based scientific and professional staff. (7,10)
**Secondary care**				
Emergency department		Non-admitted	£133.00	VB09Z Emergency medicine, category 1 investigation with category 1-2 treatment (type 1 non-admitted)[72]
		Admitted	£264.00	Weighted average of VB06Z and VB04Z[73] by severity of admission. (11)
Hospital Outpatient		Consultant visit	£204.00	CL WF01C, Non-admitted Face-to-Face attendance, Follow-up. Paediatric Respiratory Medicine. (11)
		Consultant phone	£105.00	CL WF01C, Non-admitted Non-Face-to-Face attendance, Follow-up. Paediatric Respiratory Medicine. (11)
		Nurse visit	£133.00	CHS NURS N08CF F2F, Child, Specialist Nursing, Asthma and Respiratory Nursing/Liaison, Child, Face to face. (11)

Kennedy CT, *et al. Arch Dis Child* 2024; 109:724–729. doi: 10.1136/archdischild-2023-326306

The direct and indirect cost of paediatric asthma in the UK: a cost analysis

		Nurse phone	£24.00	CHS NURS N08CF, Child. Specialist Nursing, Asthma and Respiratory Nursing/Liaison, Child, Non-face to face. (11)
		Day case	£394.00	DC, Weighted average of PD12 Paediatric Asthma or Wheezing. (11)
		Bronchoscopy	£952.00	DZ69B Diagnostic Bronchoscopy, 18 years and under, Combined day case/ordinary elective spell tariff. Admitted participant care & outpatient procedure prices 2018/19. Annex A.(12)
Hospital inpatient		Short stay (≤1 night)	£594.00	NES, Weighted average of PD12 Paediatric, Asthma or Wheezing. (11)
		Long stay	£1,913.00	NEL, Weighted average of PD12 Paediatric, Asthma or Wheezing. Inflated to 2019 prices. (7,11)
		Excess bed days	£575.00	NEL excess bed day, Weighted average of PD12 Paediatric, Asthma or Wheezing. Inflated to 2019 prices. (7,11)
Ambulance		See & treat	£209.00	AMB ASS01. (11)
		See & convey	£257.00	AMB ASS02. (11)
	Clinical psychologist		£54.00	1 hour of a clinical psychologist's time. Band 7 scientific and professional staff.(7)
	Physiotherapist		£57.00	1 hour of a specialist (respiratory problems) physiotherapist's time. Band 7 Hospital-based scientific and professional staff. (7)
	Speech and language therapist		£34.00	1 hour of a speech therapist's time. Band 5 scientific and professional staff. (7)

Kennedy CT, *et al. Arch Dis Child* 2024; 109:724–729. doi: 10.1136/archdischild-2023-326306

The direct and indirect cost of paediatric asthma in the UK: a cost analysis

Supplemental table 2: Unit cost of medication sourced from the British National Formulary for Children

Name	NHS indicative price	Pack size/ Doses	Description
Prednisolone	£31.24	28	Prednisolone 20mg tablets (A A H Pharmaceuticals Ltd)
Dexamethasone	£7.62	50	Dexamethasone 2mg tablets (A A H Pharmaceuticals Ltd)
Amoxicillin	£1.75	21	Amoxicillin 500mg capsules (A A H Pharmaceuticals Ltd)
Paracetamol (liquid)	£4.40	200	200ml Calpol Six Plus 250mg/5ml oral suspension sugar free (McNeil Products Ltd)
Paracetamol (tablet)	£1.19	16	Anadin Paracetamol 500mg tablets (Pfizer Consumer Healthcare Ltd)
Ibuprofen	£1.57	12	Nurofen 200mg tablets (Reckitt Benckiser Healthcare (UK) Ltd)
Omalizumab (75mg)	£128.07	1	Xolair 75mg/0.5ml solution for injection pre-filled syringes (Novartis Pharmaceuticals UK Ltd)
Omalizumab (150mg)	£256.15	1	Xolair 150mg/1ml solution for injection pre-filled syringes (Novartis Pharmaceuticals UK Ltd)
Theophylline	£2.96	56	Uniphyllin Continus 200mg tablets (Napp Pharmaceuticals Ltd)
Ciclosporin	£48.50	30	Capimune 100mg capsules (Mylan)
**Preventer inhaler**			
Beclomethasone	£3.70	200	Beclomethasone (Clenil) 50 mcg MDI/spacer
	£7.42	200	Beclomethasone (Clenil) 100 mcg MDI/spacer
	£16.17	200	Beclomethasone (Clenil) 200 mcg MDI/spacer
Budesonide	£14.25	200	Pulmicort 100 Turbohaler (AstraZeneca UK Ltd)
	£14.25	100	Pulmicort 200 Turbohaler (AstraZeneca UK Ltd)]
	£14.25	50	Pulmicort 400 Turbohaler (AstraZeneca UK Ltd)
Budesonide with formoterol	£28.00	120	Symbicort 100/6 Turbohaler (AstraZeneca UK Ltd)
	£28.00	120	Symbicort 200/6 Turbohaler (AstraZeneca UK Ltd)
	£28.00	60	Symbicort 400/12 Turbohaler (AstraZeneca UK Ltd)
Fluticasone	£4.00	60	Flixotide 50micrograms/dose Accuhaler (GlaxoSmithKline UK Ltd)
	£8.00	60	Flixotide 100micrograms/dose Accuhaler (GlaxoSmithKline UK Ltd)
	£6.53	120	Flixotide 50micrograms/dose Evohaler (GlaxoSmithKline UK Ltd)
	£21.26	120	Flixotide 125micrograms/dose Evohaler (GlaxoSmithKline UK Ltd)
Fluticasone with vilanterol	£22.00	30	Relvar Ellipta 92micrograms/dose / 22micrograms/dose dry powder inhaler (GlaxoSmithKline UK Ltd)

Kennedy CT, *et al. Arch Dis Child* 2024; 109:724–729. doi: 10.1136/archdischild-2023-326306

The direct and indirect cost of paediatric asthma in the UK: a cost analysis

	£29.50	30	Relvar Ellipta 184micrograms/dose / 22micrograms/dose dry powder inhaler (GlaxoSmithKline UK Ltd)
Fluticasone with formoterol	£14.40	120	Flutiform 50micrograms/dose / 5micrograms/dose inhaler (Napp Pharmaceuticals Ltd)
Fluticasone with salmeterol	£22.45	120	Aloflute 25micrograms/dose / 125micrograms/dose inhaler (Mylan)
	£17.46	60	Seretide 100 Accuhaler (GlaxoSmithKline UK Ltd)
	£33.95	60	Seretide 250 Accuhaler (GlaxoSmithKline UK Ltd)
	£32.74	60	Seretide 500 Accuhaler (GlaxoSmithKline UK Ltd)
	£17.46	120	Seretide 50 Evohaler (GlaxoSmithKline UK Ltd)
	£23.45	120	Seretide 125 Evohaler (GlaxoSmithKline UK Ltd)
	£29.32	120	Seretide 250 Evohaler (GlaxoSmithKline UK Ltd)
Ciclesonide	£38.62	120	Alvesco 160 inhaler (AstraZeneca UK Ltd)
**Reliever inhaler**			
Salbutamol	£1.50	200	Ventolin 100micrograms/dose Evohaler (GlaxoSmithKline UK Ltd)
	£3.60	60	Ventolin 200micrograms/dose Accuhaler (GlaxoSmithKline UK Ltd)
	£3.31	200	Easyhaler Salbutamol sulfate 100micrograms/dose dry powder inhaler (Orion Pharma (UK) Ltd)
	£6.30	200	Salamol 100micrograms/dose Easi-Breathe inhaler (Teva UK Ltd)
	£3.60	60	Ventolin 200micrograms/dose Accuhaler (GlaxoSmithKline UK Ltd)
Terbutaline sulfate	£8.30	120	Bricanyl 500micrograms/dose Turbohaler (AstraZeneca UK Ltd)
Ipratropium bromide	£4.14	20	Atrovent 250micrograms/1ml nebuliser liquid UDVs (Boehringer Ingelheim Ltd)
**LABA inhaler**			
Salmeterol	£29.26	120	Serevent 25micrograms/dose Evohaler (GlaxoSmithKline UK Ltd)
	£35.11	60	Serevent 50micrograms/dose Accuhaler (GlaxoSmithKline UK Ltd)
**LTRA**			
Montelukast	£25.69	28	Singulair Paediatric 5mg chewable tablets (Merck Sharp & Dohme Ltd)
	£26.97	28	Singulair 10mg tablets (Merck Sharp & Dohme Ltd)

The direct and indirect cost of paediatric asthma in the UK: a cost analysis

Supplemental table 3: Decision rules for participant's adherence score

Clinical team opinion	Smart inhaler adherence score <70%	>=70%	Not available
Adherent	- If family response is "all of the time", assume 100%<br>- If family response is "most of the time", assume 75%	- Use figure from smart inhaler	- If family response is "all of the time", assume 100%<br>- If family response is "most of the time", assume 75%
Not adherent	- Use figure from smart inhaler	- If family response is "never", assume 0%<br>- If family response is "occasionally", assume 25%<br>- If family response is "about half of the time", assume 50%	- If family response is "never", assume 0%<br>- If family response is "occasionally", assume 25%<br>- If family response is "about half of the time", assume 50%

Supplemental table 4: Healthcare resource use contacts associated with each attack stratified by highest level of care received.[1]

	All		Primary care		Secondary care	
**Number of participants (N= 244/252)[2]**	**244**		**171**		**115**	
**Number of exacerbations (N=497/509)[3]**	**497**		**291**		**160**	
	**Mean**	**SD**	**Mean**	**SD**	**Mean**	**SD**
**Total contacts per attack**	**1.5**	**1.3**	**1.4**	**1.0**	**2.0**	**1.6**
**Primary care**	**1.1**	**1.2**	1.4	**1.0**	**0.9**	**1.5**
GP	0.8	0.9	1.1	0.7	0.6	1.1
Nurse	0.1	0.5	0.1	0.4	0.2	0.6
NHS 24/111	0.1	0.4	0.1	0.4	0.1	0.3
Out of hours GP service	0.1	0.5	0.1	0.6	0.1	0.3
Walk-in centre	0.04	0.2	0.1	0.3	0.03	0.2
Pharmacist	0.0	0.06	0.0	0.06	0.01	0.1

Kennedy CT, *et al. Arch Dis Child* 2024; 109:724–729. doi: 10.1136/archdischild-2023-326306

The direct and indirect cost of paediatric asthma in the UK: a cost analysis

**Secondary care**	**0.4**	**0.6**			**1.1**	**0.4**
Emergency department	0.2	0.4			0.5	0.6
Hospital Outpatient	0.03	0.2			0.1	0.3
Hospital inpatient	0.1	0.4			0.4	0.5
Day case	0.0	0.06			0.01	0.1
Ambulance	0.02	0.2			0.1	0.3
**Medication[4]**	**1.6**	**0.7**	**1.6**	**0.7**	**1.6**	**0.8**

[1]Excludes attacks managed without contacting health service. [2]N= Patients with attacks with complete resource use data/Observed number of patients with attacks. [3]N= Attacks with complete resource use data/Observed attacks. [4] One-week course of 5mg prednisolone per day.

The direct and indirect cost of paediatric asthma in the UK: a cost analysis

Supplemental table 5: Healthcare resource use cost associated with each attack by treatment arm (N=497/509[1])

	Intervention (N=248/254)		Standard care (N=249/255)		Overall (N=497/509)	
**Number of children with at least one attack (n, %)**	**123**	**48**	**129**	**51**	**252**	**50**
**Mean number of attacks per participant among those having at least one exacerbation (mean, SD)**	**2.1**	**1.4**	**2.0**	**1.3**	**2.0**	**1.4**
**Cost per exacerbation (mean, SD)**	**£302**	**920**	**£291**	**675**	**£297**	**806**
**Primary care**	**£45**	**63**	**£39**	**35**	**£42**	**51**
GP	£33	35	£30	31	£31	33
Nurse	£2.14	8.87	£1.93	7.47	£2.03	8.19
NHS 24/111	£0.94	4.42	£1.20	5.39	£1.07	4.92
Out of hours GP service	£7.76	50	£3.57	17	£5.66	37
Walk-in centre	£1.11	8.15	£2.39	11	£1.75	9.69
Pharmacist	£0.06	0.61	£0.00	0.00	£0.03	0.43
**Secondary care**	**£256**	**925**	**£251**	**682**	**£253**	**812**
Emergency department	£19	47	£26	59	£23	54
Hospital Outpatient	£4.37	27	£4.04	26	£4.21	26
Hospital inpatient	£230	929	£211	684	£220	815
Day case	£1.59	25	£1.58	25	£1.58	25
Ambulance	£1.04	16	£8.06	49	£4.56	37
**Medication**	**£1.53**	**1.93**	**£1.77**	**1.98**	**£1.65**	**1.95**

Kennedy CT, *et al. Arch Dis Child* 2024; 109:724–729. doi: 10.1136/archdischild-2023-326306

The direct and indirect cost of paediatric asthma in the UK: a cost analysis

Supplemental table 6: Total healthcare resource use contacts observed during trial follow-up[1]

	Proportion of patients reporting any resource use (n (%))	Mean contact per patient	SD	Median contact per patient	IQR
**Healthcare contacts including follow-up visits (N=430)**	**430 (100)**	**6.7**	**4.5**	**5**	**[4, 8]**
**Healthcare contacts (N=430)**	**309 (71.9)**	**4.4**	**4.7**	**3**	**[2, 5]**
**Follow-up visits (N=506)**	**481 (95.1)**	**3.5**	**0.8**	**4**	**[3, 4]**
**Attack-related (N=498)**	**244 (49.0)**	**6.2**	**4.8**	**5**	**[3, 8]**
**Primary care**	206 (41.4)	2.7	2.6	2	[1, 3]
GP	176 (35.3)	2.3	1.9	2	[1, 3]
Nurse	28 (5.6)	2.0	1.7	1	[1, 2]
NHS 24/111	31 (6.2)	1.3	1.0	1	[1, 1]
Out of hours GP service	25 (5.0)	1.5	1.8	1	[1, 1]
Walk-in centre	15 (3.0)	1.3	0.5	1	[1, 1]
Pharmacist	2 (0.4)	1.0	0.0	1	[1, 1]
**Secondary care**	115 (23.1)	1.5	1.0	1	[1, 2]
Emergency department	67 (13.5)	1.3	0.6	1	[1, 1]
Hospital Outpatient	16 (3.2)	1.0	0.0	1	[1, 1]
Hospital inpatient	49 (9.8)	1.3	0.8	1	[1, 1]
Day case	2 (0.4)	1.0	0.0	1	[1, 1]
Ambulance	7 (1.4)	1.3	0.50	1	[1, 2]

Kennedy CT, *et al. Arch Dis Child* 2024; 109:724–729. doi: 10.1136/archdischild-2023-326306

The direct and indirect cost of paediatric asthma in the UK: a cost analysis

**Not associated with attacks (N=443)**	**218 (49.2)**	3.0	3.8	**2**	**[1, 3]**
**Primary care**	**178 (40.2)**	2.6	3.0	**2**	**[1, 3]**
GP	142 (32.1)	2.2	2.1	1	[1, 3]
Nurse	43 (9.7)	2.0	3.4	1	[1, 2]
NHS 24/111	18 (4.0)	1.4	1.2	1	[1, 1]
Out of hours GP service	15 (3.4)	1.1	0.4	1	[1, 1]
Walk-in centre	12 (2.7)	1.3	0.5	1	[1, 1.5]
Pharmacist	2 (0.5)	1.0	0.0	1	[1, 1]
**Secondary care**	**102 (23.0)**	**2.0**	**3.3**	**1**	**[1, 2]**
Emergency department	31(7.0)	1.3	0.8	1	[1, 1]
Hospital Outpatient	64 (14.4)	1.6	1.2	1	[1, 2]
Hospital inpatient	8 (1.8)	1.0	0.0	1	[1, 1]
Day case	2 (0.5)	1.0	0.0	1	[1, 1]
Bronchoscopy	4 (0.9)	1.0	0.0	1	[1, 1]
Ambulance	5 (1.1)	1.0	0.0	1	[1, 1]
Other[2]	4 (0.9)	11.0	11.5	8	[2, 20]
**Medication[3]**	**4 (0.9)**	1.0	0.0	**1**	**[1, 1.]**

[1]Results exclude 5 trial follow-up visits in secondary care (including baseline). [2]Physiotherapist/Speech and Language Therapist/Psychologist. [3]In addition to medication for asthma management prescribed at regular trial follow-up visits.

The direct and indirect cost of paediatric asthma in the UK: a cost analysis

Supplemental table 7: Total healthcare resource use cost observed during trial follow up by treatment arm[1] (N=443/506)

	Intervention (N=255)			Standard care (N=251)			Overall (N=506)		
	n	Mean	SD	n	Mean	SD	n	Mean	SD
**Total**	**223**	**£1,138**	**2790**	**207**	**£1,030**	**2160**	**430**	**£1,086**	**2504**
**Attack-related**	252	£297	1032	246	£295	854	498	£296	947
**Primary care**	252	£44	93	246	£39	66	498	£42	81
GP	252	£32	66	246	£30	55	498	£31	61
Nurse	252	£2.10	12	246	£1.96	8.09	498	£2.03	10
NHS 24/111	252	£0.93	5.09	246	£1.21	5.42	498	£1.07	5.25
Out of hours GP service	252	£7.64	51	246	£3.61	20	498	£5.65	39
Walk-in centre	252	£1.09	8.09	246	£2.42	13	498	£1.74	10.51
Pharmacist	252	£0.05	0.61	246	£0.00	0.00	498	£0.03	0.43
**Secondary care**	252	£252	1017	246	£254	855	498	£253	935
Emergency department	252	£19	56	246	£26	71	498	£23	64
Hospital Outpatient	252	£4.30	26	246	£4.09	26	498	£4.20	26
Hospital inpatient	252	£226	1012	246	£213	821	498	£220	922
Day case	252	£1.56	25	246	£1.60	25	498	£1.58	25
Ambulance	252	£1.02	16	246	£8.16	55	498	£4.55	40
Oral steroid	252	£1.51	3.79	246	£1.79	3.26	498	£1.65	3.54
**Not associated with attacks**	230	£116	247	213	£177	641	443	£145	479
**Primary care**	230	£34	69	213	£37	74	443	£35	72
GP	230	£25	58	213	£28	61	443	£26	59
Nurse	230	£4.40	24	213	£3.29	12	443	£3.87	19

Kennedy CT, *et al. Arch Dis Child* 2024; 109:724–729. doi: 10.1136/archdischild-2023-326306

NHS 24/111	230	£0.34	2.07	213	£1.22	6.55	443	£0.76	4.79
Out of hours GP service	230	£2.90	16	213	£2.78	16	443	£2.84	16
Walk-in centre	230	£0.99	6.68	213	£2.15	12	443	£1.55	9.84
Pharmacist	230	£0.00	0.00	213	£0.06	0.66	443	£0.03	0.46
**Secondary care**	230	£82	224	213	£140	635	443	£110	470
Emergency department	230	£13	62	213	£12	42	443	£12	53.
Hospital Outpatient	230	£38	90	213	£43	160	443	£40	128
Hospital inpatient	230	£8.90	79	213	£72	582	443	£39	408
Day case	230	£3.42	37	213	£0.00	0.00	443	£1.78	26
Bronchoscopy	230	£8.28	89	213	£8.94	92	443	£8.60	90
Ambulance	230	£2.23	24	213	£3.62	30	443	£2.90	27
Other[2]	230	£8.52	89	213	£0.54	7.81	443	£4.68	64
**Medication[3]**	230	£0.02	0.16	213	£0.02	0.17	443	£0.02	0.17
**Medication for asthma management**	225	£709	2364	207	£580	1753	432	£647	2092
Preventer inhaler (±LABA)	225	£290	131	207	£286	135	432	£288	133
Reliever inhaler[4]	225	£4.15	3.98	207	£3.58	2.13	432	£3.88	3.23
Separate LABA inhaler	225	£0.31	4.68	207	£1.78	26	432	£1.02	18.
LTRA	225	£20	17	207	£20	17	432	£20	17
Theophylline	225	£4.32	131	207	£4.00	12	432	£4.17	12.
Cyclosporin	225	£4.53	39	207	£4.92	41	432	£4.72	40
Omalizumab injections	225	£386	2344	207	£260	1736	432	£326	2073

[1]Results exclude 5 trial follow-up visits in secondary care (including baseline). [2]Physiotherapist/Speech and Language Therapist/Psychologist. [3]In addition to medication prescribed at regular monitoring visits for the participants asthma. [4]Assumed all patients require 4 doses per week.

The direct and indirect cost of paediatric asthma in the UK: a cost analysis

Supplemental table 8: Indirect cost of asthma captured over the trial follow-up by treatment arm (N=443/506)

	Intervention (N=228/255)		Standard care (N=213/251)		Overall (N=443/506)	
	Mean	SD	Mean	SD	Mean	SD
**Total**	**£427**	**974**	**£397**	**765**	**£412**	**879**
**Indirect costs (child)**	**£8.17**	**37**	**£10**	**37**	**£9.11**	**37**
**Indirect costs (adults[1])**	**£419**	**969**	**£387**	**753**	**£403**	**871**
Paid employment	£266	639	£249	576	£258	609
Unpaid work	£136	569	£116	334	£126	470
Leisure	£17	102	£21	109	£19	105

[1]*Where adults describes parents, friends, and relatives of the participant*

Kennedy CT, *et al. Arch Dis Child* 2024; 109:724–729. doi: 10.1136/archdischild-2023-326306

The direct and indirect cost of paediatric asthma in the UK: a cost analysis

Supplemental table 9: Results of the GLM model of direct health service costs including and excluding patients prescribed omalizumab (n=13) during trial follow-up

	Direct cost (N=430)					Direct cost (N=417)				
**Covariates**	**Predictive margins**	**SE**	**Marginal effect**	**SE**	***p*-value[1]**	**Predictive margins**	**SE**	**Marginal effect**	**SE**	***p*-value[1]**
**Predicted cost**	£1,104	250				£728	44			
**Sex**										
Male	£1,208	340				£696	57			
Female	£961	146	-£247	235	0.2	£774	52	£79.00	67	0.25
**Age**										
< 11 years old	£885	162				£701	47			
≥ 11 years old	£1,423	425	£538	303	<0.01	£762	76	£61.00	87	0.48
**Treatment level[2]**										
BTS/SIGN Step 2	£974	236				£522	113			
BTS/SIGN Step 3	£1,122	431	£148	426	0.72	£616	37	£64	99	0.55
BTS/SIGN Step 4	£1,113	153	£139	272	0.62	£864	68	£312	119	<0.05
**Number of attacks**										
0	£736	259				£356	19			
1-2	£1,138	262	£402	122	<0.05	£870	81	£514	78	<0.01
3-4	£2,302	588	£1,565	562	<0.01	£1,458	209	£1,102	213	<0.01
≥5	£2,188	434	£1,452	417	<0.01	£1,956	359	£1,600	355	<0.01
**Adherence**										
Not adherent (<70%)	£807	86				£709	55			
Adherent (≥70%)	£1,271	354	464	297	<0.05	£739	61	£30	83	0.72

[1]*p-value from GLM regression. SE: Standard Error.* [2]*Treatment levels based on the British guidelines on the management of asthma.(23) British Thoracic Society (BTS); Scottish Intercollegiate Guidelines Network (SIGN)*

Kennedy CT, *et al. Arch Dis Child* 2024; 109:724–729. doi: 10.1136/archdischild-2023-326306

The direct and indirect cost of paediatric asthma in the UK: a cost analysis

References

1. Estimates of the age distribution of parents of primary school aged children: England, Oct to Dec 2019 - Office for National Statistics [Internet]. [cited 2023 Mar 8]. Available from: https://www.ons.gov.uk/peoplepopulationandcommunity/educationandchildcare/adhocs/11865estimatesoftheagedistributionofparentsofprimaryschoolagedchildrenenglandocttodec2019

2. Earnings and hours worked, age group: ASHE Table 6 - Office for National Statistics [Internet]. [cited 2023 Mar 8]. Available from: https://www.ons.gov.uk/employmentandlabourmarket/peopleinwork/earningsandworkinghours/datasets/agegroupashetable6

3. Unpaid work calculator [Internet]. [cited 2023 Mar 8]. Available from: https://www.ons.gov.uk/visualisations/dvc376/index.html

4. GOV.UK [Internet]. 2023 [cited 2023 Mar 14]. TAG data book. Available from: https://www.gov.uk/government/publications/tag-data-book

5. GOV.UK [Internet]. [cited 2023 Mar 14]. The National Minimum Wage in 2021. Available from: https://www.gov.uk/government/publications/the-national-minimum-wage-in-2021

6. Turner S, Cotton S, Wood J, Bell V, Raja EA, Scott NW, et al. Treatment guided by fractional exhaled nitric oxide in addition to standard care in 6- to 15-year-olds with asthma: the RAACENO RCT. Efficacy and Mechanism Evaluation. 2022 Jun 6;9(4):1–154.

7. Curtis LA, Burns A. Unit Costs of Health and Social Care 2019 [Internet]. Personal Social Services Research Unit, University of Kent. Canterbury; 2019 [cited 2023 Mar 15]. 176 p. Available from: https://doi.org/10.22024/UniKent/01.02.79286

8. Hobbs FDR, Bankhead C, Mukhtar T, Stevens S, Perera-Salazar R, Holt T, et al. Clinical workload in UK primary care: a retrospective analysis of 100 million consultations in England, 2007–14. Lancet. 2016 Jun 4;387(10035):2323–30.

9. Curtis L. Unit Costs of Health and Social Care 2010 [Internet]. Personal Social Services Research Unit, University of Kent. Canterbury; 2010 [cited 2023 Jul 17]. Available from: https://www.pssru.ac.uk/pub/uc/uc2010/uc2010.pdf

10. Pope C, Turnbull J, Jones J, Prichard J, Rowsell A, Halford S. Has the NHS 111 urgent care telephone service been a success? Case study and secondary data analysis in England. BMJ Open. 2017 May 1;7(5):e014815.

11. NHS England » 2018/19 National Cost Collection Data Publication [Internet]. [cited 2023 Jul 17]. Available from: https://www.england.nhs.uk/publication/2018-19-national-cost-collection-data-publication/

12. National Tariff 2017/19: documents and policies [Internet]. NHS Improvement; 2021 [cited 2023 Jul 17]. Available from: https://www.england.nhs.uk/wp-content/uploads/2021/02/2017-19_NTPS.zip

Kennedy CT, *et al. Arch Dis Child* 2024; 109:724–729. doi: 10.1136/archdischild-2023-326306
